# Association between ICU admission during off-hours and in-hospital mortality: a multicenter registry in Japan

**DOI:** 10.1186/s40560-022-00634-3

**Published:** 2022-09-05

**Authors:** Yu Namikata, Yoshinori Matsuoka, Jiro Ito, Ryutaro Seo, Yasukazu Hijikata, Takahiro Itaya, Kenjiro Ouchi, Haruka Nishida, Yosuke Yamamoto, Koichi Ariyoshi

**Affiliations:** 1 Department of Emergency Medicine, Kobe City Medical Center General Hospital, 2-1-1 Minatojima-Minamimachi, Chuo-Ku, Kobe, Hyogo 650-0047 Japan; 2 Department of Healthcare Epidemiology, Graduate School of Medicine and Public Health, Kyoto University, Yoshidakonoe-Cho, Sakyo-Ku, Kyoto, 606-8501 Japan; 3 Department of Anesthesia and Critical Care, Kobe City Medical Center General Hospital, 2-1-1 Minatojima-Minamimachi, Chuo-Ku, Kobe, Hyogo 650-0047 Japan

**Keywords:** After-hours care, Intensive care units, Hospital mortality, Health services research, Time of admission

## Abstract

**Background:**

The effect of ICU admission time on patient outcomes has been shown to be controversial in several studies from a number of countries. The imbalance between ICU staffing and medical resources during off-hours possibly influences the outcome for critically ill or injured patients. Here, we aimed to evaluate the association between ICU admission during off-hours and in-hospital mortality in Japan.

**Methods:**

This study was an observational study using a multicenter registry (Japanese Intensive care PAtient Database). From the registry, we enrolled adult patients admitted to ICUs from April 2015 to March 2019. Patients with elective surgery, readmission to ICUs, or ICU admissions only for medical procedures were excluded. We compared in-hospital mortalities between ICU patients admitted during off-hours and office-hours, using a multilevel logistic regression model which allows for the random effect of each hospital.

**Results:**

A total of 28,200 patients were enrolled with a median age of 71 years (interquartile range [IQR], 59 to 80). The median APACHE II score was 18 (IQR, 13 to 24) with no significant difference between patients admitted during off-hours and those admitted during office-hours. The in-hospital mortality was 3399/20,403 (16.7%) when admitted during off-hours and 1604/7797 (20.6%) when admitted during office-hours. Thus, off-hours ICU admission was associated with lower in-hospital mortality (adjusted odds ratio 0.91, [95% confidence interval, 0.84–0.99]).

**Conclusions:**

ICU admissions during off-hours were associated with lower in-hospital mortality in Japan. These results were against our expectations and raised some concerns for a possible imbalance between ICU staffing and workload during office-hours. Further studies with a sufficient dataset required for comparing with other countries are warranted in the future.

## Background

Critically ill patients in intensive care units (ICUs), suffering from a variety of organ disorders, require prompt diagnosis and treatment [1]. It has been advocated that what is done by healthcare professionals during the first hour in the ICU is of the utmost importance [2–4]. However, we might not have an adequate number of medical staff during off-hours such as weekday nightshifts and weekends. Such a lack of medical resources often leads to delays in diagnostic and treatment procedures, which, unfortunately, might be postponed until office-hours [5]. Naturally, concerns have been raised about the association between poor patient outcomes and the imbalance between demand and supply of medical resources during off-hours.

To date, a number of studies have been carried out to address long-standing concerns as to whether admission to the ICU during off-hours would affect patient outcomes. Some studies have demonstrated that ICU admission during weekday nightshifts or holidays were associated with worse outcomes in patients admitted to ICUs [5–11], while others showed that mortality was similar between patients admitted during off-hours and those admitted during office-hours [4, 12–20]. It is believed that this inconsistency in the findings may be explained by multifactorial reasons, such as differences in the definition of office-hours, differences in study population, study size or insufficient correction for case mix and severity of illness [5]. Furthermore, we should also be cautious about variations in clinical practice and organizational structures in each country [21, 22]. As for Japan, there have been no domestic clinical trials or multicenter observational studies, so it remains unclear whether or not ICU admission time affects a patient’s outcome under the current medical system.

The purpose of this study was to evaluate the association between ICU admission during off-hours and in-hospital mortality in Japan, and to provide useful information for administrators and health policymakers when considering appropriate medical staffing in ICUs and the efficient allocation of other medical resources.

## Methods

### Study design and data collection

This was a multicenter observational study based on the national ICU registry, the Japanese Intensive care PAtient Database (JIPAD), which the Japanese Society of Intensive Care Medicine launched in 2014, and prospectively collected individual patient data and information on the participating facility in a predefined manner [23]. As of 2017, a total of 21 ICUs with 217 beds had participated in the registry.

The following patient information was registered in the JIPAD: age, sex, height, weight, date and time of admission, admission source (emergency department, operating room, ward, other care units, or transferred from other hospitals), type of admission (elective, emergency, and ICU medical procedure), diagnosis at ICU admission (cardiovascular disease, respiratory disease, gastrointestinal disease, metabolic disease, neurological disease, hematological disease, musculoskeletal disease, obstetric and gynecological disease, genitourinary disease, trauma, etc.), after cardiac resuscitation, Acute Physiology and Chronic Health Evaluation (APACHE) II score [24], and chronic organ insufficiencies (acquired immunodeficiency syndrome, heart failure, respiratory failure, liver cirrhosis, acute leukemia or multiple myeloma, lymphoma, metastatic cancer, immunosuppression, and maintenance dialysis). We also extracted the following facility-level information: number of intensivists dedicated to ICUs, number of full-time nurses designated to ICUs, number of ICU beds, number of hospital beds and type of hospital (university, public, or private).

### Selection of participants

Out of all the patients in the JIPAD registry, we enrolled adult patients aged 18 years and older from April 2015 to March 2019. Referring to previous studies, we excluded those who were admitted to ICUs after elective surgery, or who were readmitted to ICUs, or who were admitted to ICUs for ICU clinical procedures and discharged alive within 24 h [4, 5, 9, 14, 18, 19]. Further, we excluded patients with missing data on ICU admission time, in-hospital mortality, or covariates for statistical analysis.

### Off-hours vs. office-hours

We defined office-hours as from 09:00 to 17:00, weekdays, Monday to Friday, and regarded official public holidays and all other times as off-hours. Although there is no standard definition of office-hours, we referred to previous studies and finally adopted the practical definition of office-hours and off-hours as above, which has been commonly adopted in Japan as a practical approach [5, 12].

### Outcome measures

We defined in-hospital mortality as the outcome of interest, and compared the outcome between two groups—an off-hours group and an office-hours group.

### Statistical analysis

We described continuous variables with medians and interquartile ranges (IQRs), and categorical variables with counts and proportions. We examined continuous variables using Mann–Whitney *U* test, and categorical variables using the Chi-square test.

### Primary analysis

In the primary analysis, we compared patient outcomes between ICU patients admitted during off-hours and office-hours, using a multilevel logistic regression model which allows for the random effect of each hospital (a random-intercept model).

In this model, we used a complete data set and adjusted both patient-level variables and facility-level variables as follows: age, sex, body mass index (< 18.5, 18.5 to 25, ≥ 25) [25], APACHE II score, the most common three diagnoses at ICU admission (cardiovascular disease, respiratory disease, and neurological disease), trauma, surgery, after cardiac resuscitation, admission source (emergency department, operating room, ward, other care units, or transferred from other hospitals), number of intensivists in relation to the number of ICU beds, number of full-time ICU nurses in relation to the number of ICU beds, number of hospital beds (categorized into tertiles) and type of hospital (university, public, or private). Further, we conducted a sensitivity analysis for missing covariates with multivariate imputation by chained equations. To impute the missing data, we used all measured variables, including outcomes, and generated 20 imputed data sets based on the assumption that the data were missing randomly.

### Additional analysis

In order to evaluate the associations between the ICU admission time and in-hospital mortality in detail, we calculated predicted in-hospital mortality for each ICU admission time (every hour from 0:00 to 23:00). Initially, we divided patients into those who were admitted during weekdays and weekends (including official public holidays). We then estimated in-hospital mortality for both of these groups using a multilevel logistic regression model, which adjusted the same variables as the primary analysis and allowed for each hospital as a random effect.

### Subgroup analysis

In order to evaluate the heterogeneity of the target population in the present study, we decided to perform an a priori subgroup analysis. In the subgroup analysis, we took into consideration patient factors (disease categories and requirement of surgery) and facility factors (the number of intensivists dedicated to ICUs, the number of full-time ICU nurses, and type of hospital) as potential sources of heterogeneity. Here, we adopted the most prevalent diseases (cardiovascular, respiratory and neurological disorder) as the subgroups of the disease categories [11].

We conducted all statistical analyses using STATA version 16.1 (StataCorp, College Station, TX), and all hypothesis tests were 2-tailed with a significance level of *P* < 0.05.

The ethics committee of Kobe City Medical Center General Hospital approved this study protocol (zn200717). The JIPAD project itself was approved by the ICU Functional Assessment Committee of the Japanese Society of Intensive Care Medicine. We conducted the present study with an opt-out policy from patients, their relatives, or proxies, and written informed consent was waived.

## Results

### Study population

**Fig. 1:**
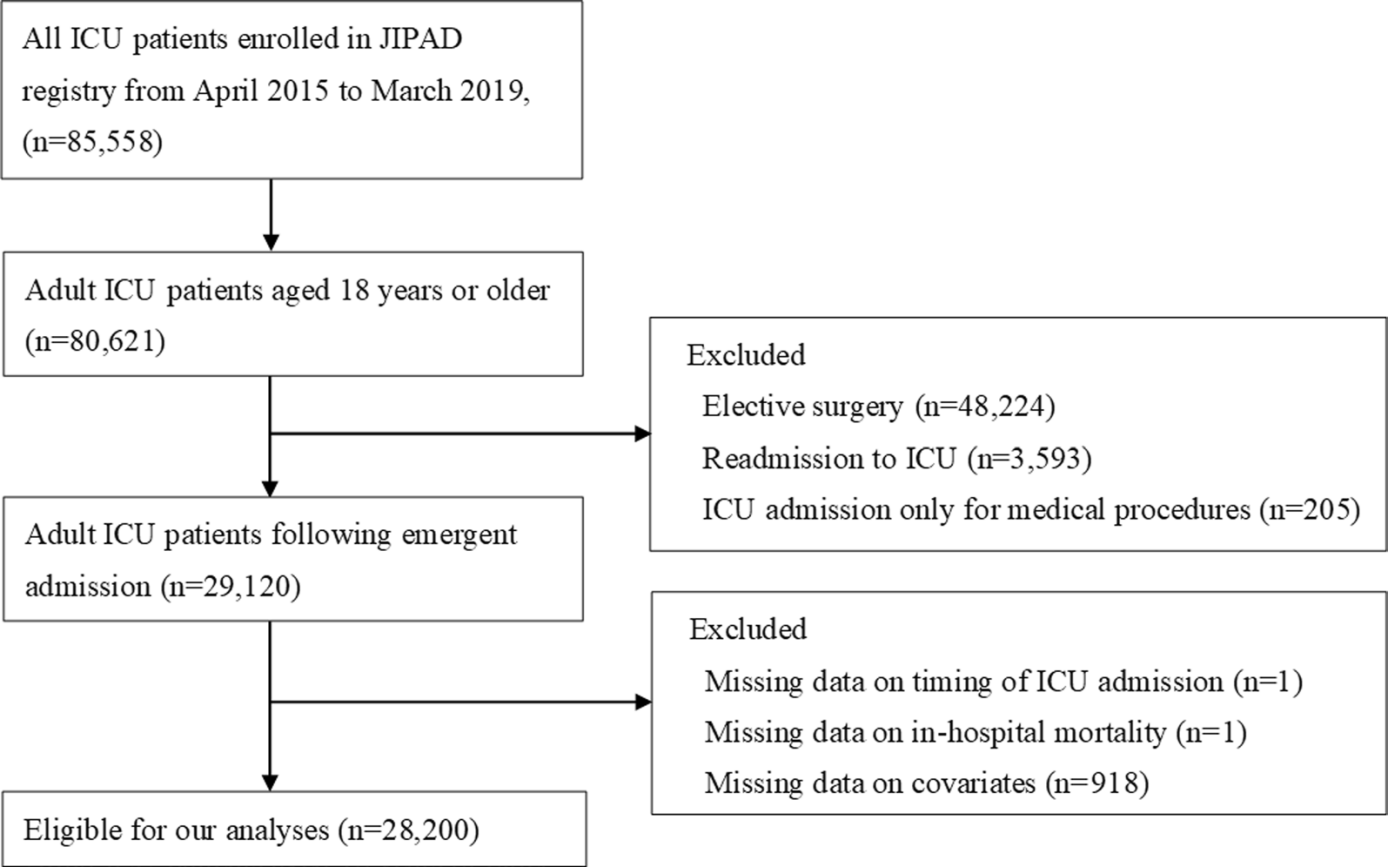
Flowchart of the study. For the analysis, we precluded cases where data on the variables for the multilevel logistic model were missing. *ICU* intensive care unit, *JIPAD* Japanese Intensive care PAtient Database

During the study period, a total of 85,558 patients were enrolled in the JIPAD registry, and 28,200 patients were eligible for analysis in our study (Fig. 1). We excluded patients who were less than 18 years old (4937), patients who had elective surgeries (48,224), readmissions (3593), and patients who stayed in ICUs only for medical procedures (205). Patients with missing data on ICU admission time, in-hospital mortality, or covariates for the statistical analysis were excluded (920).

### Patient characteristics

Table 1 shows the baseline characteristics of patients who were admitted to ICUs during off-hours and office-hours.

**Table 1:** Clinical characteristics and outcomes of patients admitted to ICUs during off-hours vs. office-hours

	Total (*n* = 28,200)	Off-hour admission (*n* = 20,403)	Office-hour admission (*n* = 7797)
Age, median (IQR)	71 (59–80)	71 (59–80)	71 (60–80)
Female, *n* (%)	10,902 (38.7)	7986 (39.1)	2916 (37.4)
BMI, median (IQR)	22.2 (19.6 to 25.0)	22.2 (19.5 to 24.9)	22.3 (19.6 to 25.2)
APACHE II score, median (IQR)	18 (13 to 24)	17 (12 to 24)	18 (13 to 25)
Admission source, *n* (%)			
Emergency departments	13,588 (48.2)	9751 (47.8)	3837 (49.2)
Operating rooms	7689 (27.3)	6465 (31.7)	1224 (15.7)
Non-ICU wards	5739 (20.4)	3611 (17.7)	2128 (27.3)
Transfer from other hospitals	617 (2.2)	273 (1.3)	344 (4.4)
Other	567 (2.0)	303 (1.5)	264 (3.4)
Type of hospital, *n* (%)			
National/public hospitals	9564 (33.9)	6780 (33.2)	2784 (35.7)
University hospitals	11,694 (41.5)	8453 (41.4)	3241 (41.6)
Private hospitals	6942 (24.6)	5170 (25.3)	1772 (22.7)
Diagnosis at ICU admission, *n* (%)			
Cardiovascular	9839 (34.9)	6848 (33.6)	2991 (38.4)
Respiratory	3531 (12.5)	2315 (11.3)	1216 (15.6)
Neurologic	4105 (14.6)	3079 (15.1)	1026 (13.2)
Trauma	1575 (5.6)	1227 (6.0)	348 (4.5)
Emergent surgery, *n* (%)	9517 (33.7)	7647 (37.5)	1870 (24.0)
After cardiac resuscitation, *n* (%)	1713 (6.1)	1178 (5.8)	535 (6.9)
Chronic organ insufficiency, *n* (%)			
Liver cirrhosis	602 (2.1)	420 (2.1)	182 (2.3)
Renal dialysis	1638 (5.8)	1128 (5.5)	510 (6.5)
Hematologic malignancy	652 (2.3)	416 (2.0)	236 (3.0)
Solid tumor with metastasis	916 (3.2)	681 (3.3)	235 (3.0)
Immunosuppression	1828 (6.5)	1,231 (6.0)	597 (7.7)
One or more insufficiencies	5526 (19.6)	3819 (18.7)	1707 (21.9)
Invasive interventions in ICUs, *n* (%)			
Mechanical ventilation	13,234 (46.9)	9539 (46.8)	3695 (47.4)
CRRT	1890 (6.7)	1273 (6.2)	617 (7.9)
IABP	1131 (4.0)	750 (3.7)	381 (4.9)
ECMO	556 (2.0)	378 (1.9)	178 (2.3)
In-hospital mortality, *n* (%)	5003 (17.7)	3399 (16.7)	1604 (20.6)

*APACHE II* Acute Physiology and Chronic Health II, *BMI* body mass index, *CRRT* continuous renal replacement therapy, *ECMO* extracorporeal membrane oxygenation, *IABP* intra-aortic balloon pump, *ICU* intensive care unit, *SD* standard deviation

The median age was 71 (IQR, 59–80) years, and 38.7% of patients were female. The median APACHE II score was 18 (IQR, 13–24), and this was comparable between patients who were admitted during off-hours and office-hours (17 vs. 18, respectively).

ICU admissions from operating rooms were more common during off-hours than during office-hours (31.7 vs. 15.7%, respectively). Conversely, patients admitted to ICUs from non-ICU wards, or who had been transferred from other hospitals, were more frequently observed during office-hours than during off-hours. There seemed no clinically important differences in the type of hospital between the two groups.

Among the diagnoses at ICU admission, neurologic diseases were more likely to be the reason for admission during off-hours, while cardiovascular diseases and respiratory diseases were more common during office-hours. Additionally, emergent surgeries were performed in about one-third of the total number of patients, and were more often performed among patients who were admitted during off-hours than those admitted during office-hours (37.5% vs. 24.0%, respectively).

With regard to chronic organ insufficiencies, ICU patients admitted during off-hours were less likely to have one or more insufficiencies compared with those admitted during office-hours (18.7 vs. 21.9%, respectively), but there were no clinically important differences in distribution among those with chronic organ insufficiencies.

Invasive interventions, such as mechanical ventilation, continuous renal replacement therapy, intra-aortic balloon pumping and extracorporeal membrane oxygenation were more likely to be performed in ICU patients who were admitted during office-hours.

### In-hospital mortality∶ off-hours vs. office-hours

**Fig. 2:**
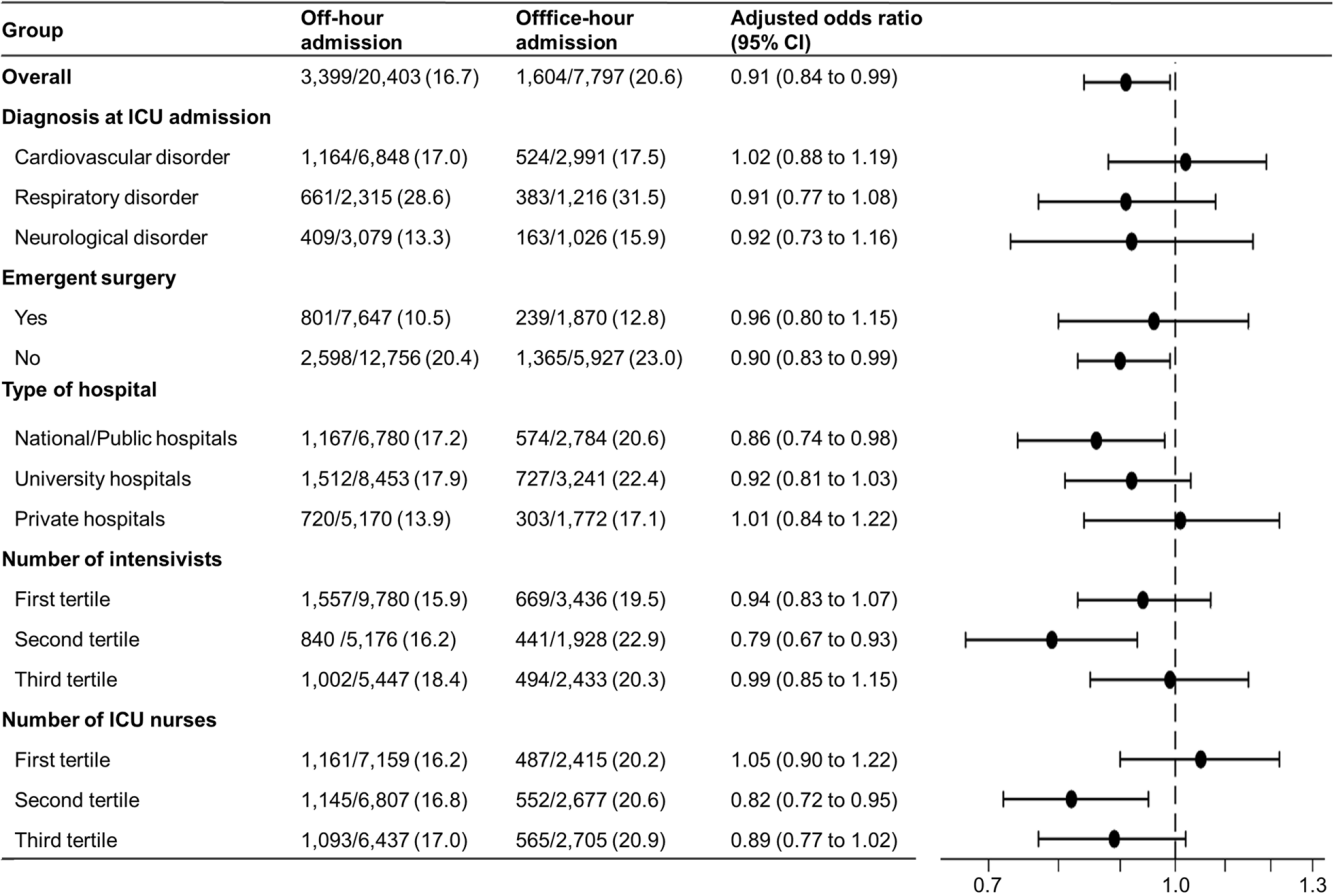
In-hospital mortality and ICU admission time in all patients and subgroups, stratified by diagnosis at ICU admission, emergency surgery, and facility factors: off-hours vs. office-hours. Office-hours were defined as being from 09:00 to 17:00, weekdays, Monday to Friday, with official public holidays and all other times regarded as off-hours. Adjusted odds ratios were calculated using a multilevel logistic regression model, allowing for each hospital as a random effect (a random-intercept model). Here, we adjusted both patient-level variables and facility-level variables as follows: age, gender, BMI (< 18.5, 18.5 to 25, ≥ 25), APACHE II score, the most common three diagnoses at ICU admission (cardiovascular disease, respiratory disease, and neurological disease), trauma, emergent surgery, after cardiac resuscitation, admission source (emergency department, operating room, ward, other care units, or transferred from other hospitals), the number of intensivists in relation to the number of ICU beds, the number of ICU nurses in relation to the number of ICU beds, the number of hospital beds (categorized into tertiles), and the type of hospital (university, public or private). *APACHE* Acute Physiology and Chronic Health Evaluation, *BMI* body mass index, *CI* confidence interval; ICU, intensive care unit

Figure 2 demonstrates in-hospital mortality and ICU admission time in all patients and the predefined subgroups, stratified by diagnosis at ICU admission, emergency surgery, and facility factors.

The in-hospital mortality was significantly lower in patients admitted to ICUs during off-hours than in those admitted during office-hours (3399/20,403 (16.7%) vs. 1604/7797 (20.6%); adjusted odds ratio, 0.91 [95% confidence interval, 0.84 to 0.99]). The sensitivity analysis, using the imputed data set, revealed similar results to the main analysis, with an unadjusted odds ratio of 0.78 (95% confidence interval: 0.73–0.83) and adjusted odds ratio of 0.89 (95% confidence interval: 0.82–0.97).

### Predicted in-hospital mortality at the time of ICU admission

**Fig. 3:**
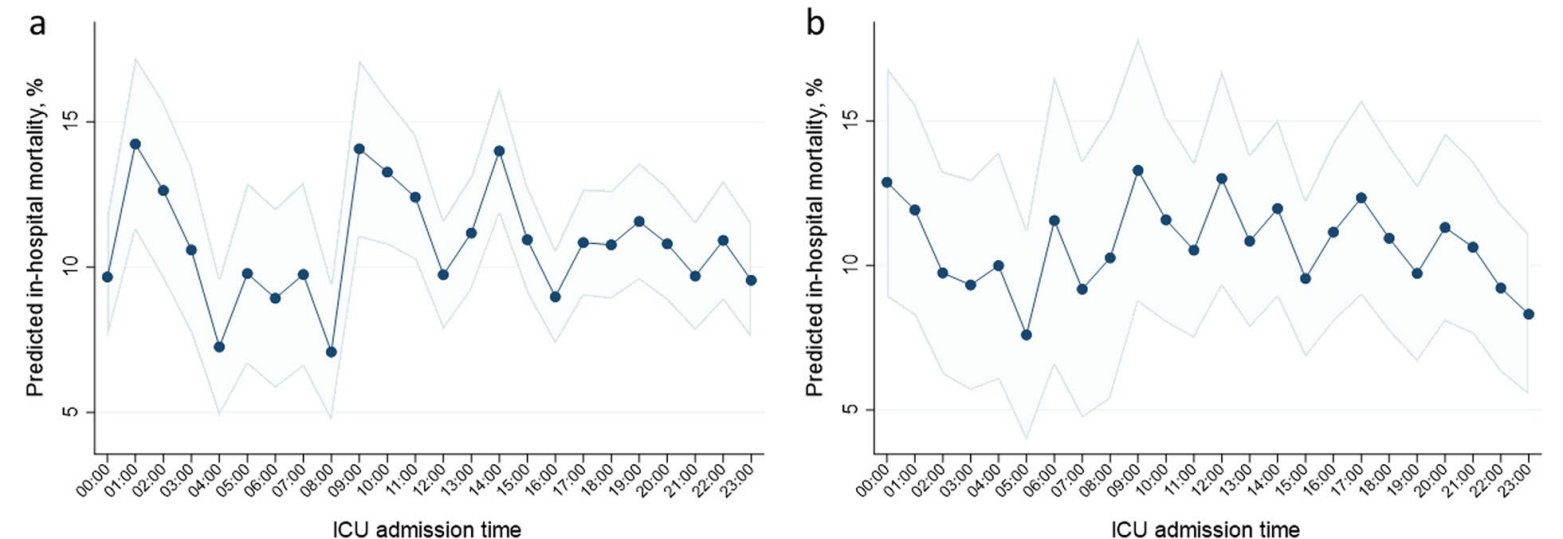
Predicted in-hospital mortality at each time of ICU admission. We calculated predicted in-hospital mortality with 95% CIs at each time of ICU admission during weekday (**a**) and weekend (**b**). Here, we adopted a multilevel logistic regression model which adjusted both patient-level variables and facility-level variables and allowed for each hospital as a random effect. *CI* confidence interval, *ICU* intensive care unit

The predicted in-hospital mortality at each time of ICU admission was calculated from the statistical model, and revealed diurnal fluctuation only on weekdays, but not at weekends (Fig. 3). On weekdays, the predicted in-hospital mortality demonstrated diurnal fluctuation: it formed three peaks, a steep rise from 08:00 to 09:00 and two peaks at 14:00 and 01:00. Conversely, weekend predicted in-hospital mortality was relatively stable, without forming similar peaks that were observed on weekdays.

### Subgroup analysis

In the subgroup analysis, there appeared to be no clinically important heterogeneity due to disease category (cardiovascular disorder, neurological disorder, trauma, and others) or facility factors (number of certified intensivists, number of full-time ICU nurses, and ICU beds).

## Discussion

In this review of a multicenter registry in Japan, ICU patients were likely to develop lower in-hospital mortality when they were admitted during off-hours than during office-hours in our domestic setting. Additionally, predicted in-hospital mortality at each time period for ICU admission showed a diurnal fluctuation only during weekdays—a steep rise from 08:00 to 09:00 with two further peaks. In the subgroup analysis, the apparent heterogeneity in the association between ICU admission during off-hours and in-hospital mortality was not clearly indicated in each individual group.

There has been a discrepancy in the results of studies evaluating the association between patient outcomes and time of ICU admission [15]. Indeed, while some previous studies reported that patient outcomes were similar between those who were admitted during off-hours and those admitted during office-hours [4, 12–20], others demonstrated that off-hours admission to the ICU led to poor patient outcomes [5–11]. Further, against our expectations, there have been two observational studies that demonstrated that office-hours admission to ICUs was associated with poor patient prognosis [16, 20]: one was a large multicenter observational cohort which was conducted in France [16], and the other was a single-center retrospective observational study conducted in the United States [20]. While both of these previous studies assessed the confounding factors with a logistic model using patient-level variables alone, we added facility-level factors into the model, and also took variance in each facility into consideration by using the multilevel logistic model. However, we reached the same conclusions as the two previous studies. Based on the results of the present study and these previous reports, although this association appears to be surprising intrinsically, the negative influence of office-hours on admission to the ICU might occur under certain specific settings. However, we should evaluate these findings with caution in that observations based on Japanese data may not apply to other countries. In addition, we believe that an international comparison is helpful to identify organizational factors responsible for this association, and further studies with a sufficient dataset are warranted in the future.

Some hypotheses have been proposed suggesting that there are mechanisms underlying the association between poor in-hospital mortality and ICU admission during office-hours [16, 26–29]. First, previous studies advocated the concept of a so-called “inverse weekday effect” that might stem from under-resourced needs, such as the lack or unavailability of specialists or operating rooms [30]. During regular times, it is possible that there could be competition for medical personnel and resources between emergency and scheduled patients, such as those who are admitted after elective surgeries. Second, an increase in the daily workload might have an adverse effect on the quality of patient care provided by healthcare workers in the ICU. For example, during office-hours, ICU nurses have to manage a large daily workload, such as dealing with new requests and instructions, caring for ICU patients, transporting stabilized patients to other wards, and so forth [26–29]. Furthermore, intensivists also have to manage an increased workload during the daytime. They are required to perform a large amount of work in addition to patient care and medical check-ups, such as doing ICU rounds, giving an explanation of the patient’s medical condition to their family, and teaching and lecturing ICU residents [16]. Third, fatigue and a subsequent lack of attention might play an unintentional role in the increase in developing complications [16]. Intensivists might get fatigued because they have to perform invasive diagnostic tests and provide treatment with a high risk of fatal complications more commonly during office-hours.

Further, in our additional analyses, an apparent diurnal fluctuation was observed in the predicted in-hospital mortality only during weekdays but not at weekends. Such findings could indicate that the mechanism of these findings might be related to staff shifts and regular work during weekdays. In addition, we demonstrated that in-hospital mortality showed a steep rise from 08:00 to 09:00, which suggested the possibility of the risk being highest around the changeover from the night shift to the day shift.

Unfortunately, based on the present study and current evidence, we could not draw any conclusive ideas on the mechanisms underlying this association. Regarding the nature of the database in the present study, sufficient hospital-level information, which is required to interpret the findings, is not available. However, as such notions regarding the influence of ICU staffing and workloads on patient outcomes are, rationally speaking, plausible, further studies will be required in order to evaluate possible problems caused by these organizational factors.

This study has the advantage of using a nationwide multicenter database, JIPAD, which we believe is suitable for evaluating the actual situation of ICUs in Japan. Furthermore, this particular database holds records of a variety of both patient-level factors and facility-level factors. In order to consider variance among facilities, we adopted a multilevel regression model, clustering patients within each hospital, and were able to adjust both patient-level and facility-level potentially confounding factors.

### Limitations

There are several limitations that may affect the interpretation of this observational study. First, unfortunately, we did not have sufficient information on medical staffing during each shift, nor on the organizational characteristics of the ICUs. For example, in order to speculate what the underlying mechanism of these unexpected results is, we need detailed data on such factors as the number and the amount of experience of the intensivists working in the various ICUs, patient-to-nurse ratios during office-hours and off-hours, and 24-h coverage by intensivists in each of the ICUs. Further studies are required using multicenter databases that possess such detailed organizational information. Second, as a previous study pointed out, there might be the possibility of misclassifications of office-hours and off-hours [31] and, furthermore, there might not even be a general consensus on the definition of office-hours and off-hours. We performed the additional analysis without using such a definition, but calculated predicted in-hospital mortality for each time period of ICU admission. In the additional analysis, we observed that there was a diurnal fluctuation in the association between in-hospital mortality and the time of ICU admission, though mortality tended to be higher during office-hours. Third, we were not able to fully speculate what the mechanism of such unexpected results was. We hypothesized a priori that there had been heterogeneity in this target population and the facilities concerned, and that this would be associated with any such mechanism. However, in the subgroup analysis, we could not find any apparent heterogeneity. Fourth, we should evaluate any influences from outside the ICU, which might be beyond the scope of the present study. Indeed, several studies have reported the impact of boarding time in emergency departments or time of surgery on patient outcomes [32, 33]. However, unfortunately, we did not have any patient data from emergency departments or operating rooms. Fifth, generalizability to other countries was limited because this is a multicenter observational cohort study in Japan. It is difficult to extrapolate the results of the present study to other countries and settings with different medical systems, resources, and organizations. Sixth, we should consider the influence of unmeasured and residual confounding. Unmeasured confounding includes, for example, competition for medical personnel and resources during office-hours, and differences in quality of care among ICUs with varying degrees of actively accepting patients during off-hours. Further, we should interpret the present findings with caution as there may be residual confounding via severity of illness, not sufficiently measured from the APACHE II score. For instance, patients admitted to ICU during office-hours may be clinically more severe than those admitted during off-hours. Some critically ill patients in the nighttime might hesitate or withdraw from aggressive treatment and admission to ICUs. Thus, admission to ICU during off-hours might be selected conservatively. On the other hand, in the daytime, even if patients are challenging, they might tend to be admitted to ICUs. Finally, we believe that selection bias exists as the facilities enrolled in the JIPAD might be resource-rich and capable of providing advanced medical management 24 h a day, 7 days a week.

## Conclusions

In this review of a multicenter registry in Japan, ICU patients were likely to develop lower in-hospital mortality when they were admitted during off-hours than during office-hours in the domestic setting. Although there remain many unclear factors with regard to the mechanism, we should pay attention to the potential imbalance between ICU staffing and their workload during office-hours. Further studies are warranted to investigate both the clinical implications and the underlying mechanism of these findings in order to develop ICU systems with an appropriate distribution of medical resources and to provide better intensive care regardless of admission time.

## Data Availability

Data from JIPAD are not allowed to be published or to be shared with third parties under the author’s agreement with JIPAD. However, details regarding the analytic methods used in the study are available from the corresponding author upon reasonable request. The JIPAD Working Group would cooperate in the case of any fraudulent use or plagiarism being suspected with regard to manuscripts using data sets from JIPAD.

## Abbreviations

APACHE: Acute Physiology and Chronic Health Evaluation
BMI: Body mass index
CI: Confidence interval
CRRT: Continuous renal replacement therapy
ECMO: Extracorporeal membrane oxygenation
IABP: Intra-aortic balloon pump
ICU: Intensive care unit
JIPAD: Japanese Intensive care Patient Database
SD: Standard deviation
